# Anthropozoonotic Endoparasites in Free-Ranging “Urban” South American Sea Lions (*Otaria flavescens*)

**DOI:** 10.1155/2016/7507145

**Published:** 2016-03-09

**Authors:** Carlos Hermosilla, Liliana M. R. Silva, Mauricio Navarro, Anja Taubert

**Affiliations:** ^1^Institute of Parasitology, Justus Liebig University Giessen, 35392 Giessen, Germany; ^2^Institute of Pathology, University Austral of Chile, Valdivia, Chile; ^3^University of California Davis School of Veterinary Medicine, Sacramento, CA 95616, USA

## Abstract

The present study represents the first report on the gastrointestinal endoparasite fauna of a free-ranging “urban” colony of South American sea lions (*Otaria flavescens*) living within the city of Valdivia, Chile. A total of 40 individual faecal samples of South American sea lions were collected during the year 2012 within their natural habitat along the river Calle-Calle and in the local fish market of Valdivia. Coprological analyses applying sodium acetate acetic formalin methanol (SAF) technique, carbol fuchsin-stained faecal smears and* Giardia*/*Cryptosporidium* coproantigen ELISAs, revealed infections with 8 different parasites belonging to protozoan and metazoan taxa with some of them bearing anthropozoonotic potential. Thus, five of these parasites were zoonotic (Diphyllobothriidae gen. sp., Anisakidae gen. sp.,* Giardia*,* Cryptosporidium*, and* Balantidium*). Overall, these parasitological findings included four new parasite records for* Otaria flavescens*, that is,* Giardia*,* Cryptosporidium*,* Balantidium*, and* Otostrongylus.* The current data serve as a baseline for future monitoring studies on anthropozoonotic parasites circulating in these marine mammals and their potential impact on public health.

## 1. Introduction

The South American sea lions (*Otaria flavescens*, Carnivora: Otariidae) are common pinnipeds living along the eastern and western coasts of South America and are generally found in Peru, Chile, Argentina, and South Brazil [1–6]. Along the Chilean coastal shores, more than 200 colonies of free-ranging sea lions have been described. A vast amount of data on feeding ecology, reproduction, life history parameters, and population dynamics of these otariid species was published [7–16]. Several studies have also addressed aspects of the helminth fauna of South American sea lions, comprising single species records, taxonomy, and population studies of some parasitoses [5, 14–22]. However, very little is known on protozoan parasite infections of these free-ranging marine mammals.

Although their natural habitat is the marine environment, several pinniped and cetacean species are found in rivers containing fresh water. As such, a stable population of South American sea lions has been established within the city of Valdivia, Chile, resulting in permanent colonization for 20 years now. These animals have adapted extremely well not only to the fresh water of the river but also to human activities in the river Calle-Calle, such as regular ship- and boat-trafficking, rowing, kayaking, and sealing activities. This “urban” sea lion colony is allocated approximately 7 km upstream from the ocean shore and animals mainly feed on fish captured by themselves in the river (mainly carps, trouts, and salmons) or on remains of the local fish market. This unusual urban colony of South American sea lions consists of more than 72 individuals and is exclusively composed of males. The age of these animals varies from 2 to 15 years but some animals might be even older. Given that* O. flavescens* need to rest after swimming and diving activities, the sea lions in Valdivia utilize river floats, riverside piers, and footways along the river promenade as recreation areas with all of them being allocated in close proximity to inhabitants, tourists, domestic pets, or the local fish market. Since some of these animals behave rather aggressive towards humans, animals, or even vehicles and additionally tend to expand their territory into the city center, the local city authorities have established a “sea lion task force” which should prevent the animals from harming humans and withhold them to premises alongside the river shore. However, given that these animals nowadays represent a tourist attraction, direct contacts of humans with these animals or their faeces as well as faecal contamination of the river water or the terrestrial environment cannot be avoided.

The present study therefore aimed to identify the actual gastrointestinal fauna in these free-ranging sea lions within their natural habitat in the river of the city of Valdivia, Chile, and to gain some insights in their potential zoonotic impact on public health issues.

## 2. Material and Methods

### 2.1. Study Area, Sample Collection, and Coproscopical Analyses

**Figure 1:**
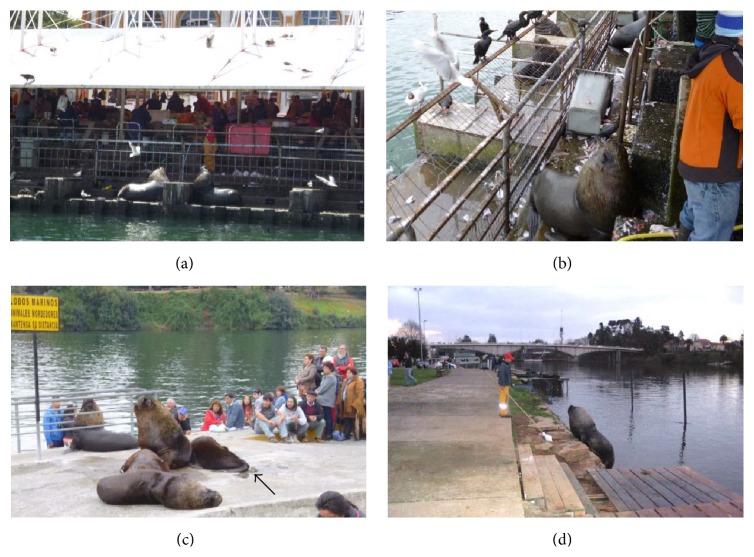
Illustration of urban South American sea lions (*Otaria flavescens*) in the city of Valdivia, Chile. (a) Sea lions on river shore of the fish market; (b) sea lions within the fish market premise in close contact to fisherman and sea products; (c) a group of sea lions at the promenade of the river surrounded by tourists despite the yellow signpost indicating the danger to get bitten by these wild animals (arrow indicates faecal contamination); (d) officer of the local “sea lion task force” using a stick with plastic bag fixed on the tip frightening an aggressive sea lion male to keep out of the promenade.

South American sea lions (*O. flavescens*) were studied along the shores of the river Calle-Calle of the city of Valdivia, Chile. The study area encompassed 3 km^2^ and comprised river floats, riverside piers, footways along the river promenade, and the local fish market with all of them being allocated in close proximity of humans and domestic pets (Figures 1(a), 1(b), 1(c), and 1(d)). A total of 40 individual faecal samples were collected during the summer of 2012. Whenever defecation occurred, scat samples were immediately collected and transferred into 2 mL plastic tubes (Eppendorf). The samples were fixed in 70% ethanol and stored at 4°C until further analysis. Parasitological analyses were conducted at the Institute of Parasitology, Justus Liebig University Giessen, Germany. Coproscopical analyses were performed by using the standard sodium acetate acetic acid formalin (SAF) technique [23]. The SAF technique was used for the detection of parasite eggs, cysts, sporocysts, and oocysts within faecal material in marine mammals as described elsewhere [24]. Additionally, a carbol fuchsin-stained faecal smear (CFS) [25] was carried out for the detection of* Cryptosporidium* oocysts. Moreover, coproantigen ELISAs (ProSpecT®, Oxoid) were performed for the detection of* Cryptosporidium* and* Giardia* antigens in faecal samples. The parasitological identification of eggs and cysts was based on morphological criteria referring to previous reports [24, 26, 27]. All sampling procedures were conducted in accordance with Institutional Ethic Commission of University Austral of Chile and the current Chilean Animal Law.

### 2.2. Molecular Analyses of Giardia-Positive Samples

*Giardia*-positive sea lion samples were further analyzed for the presence of* G. intestinalis* DNA by conventional and nested PCR detecting the beta-giardin gene (assemblage C). Genomic DNA was extracted from sea lion faecal material using the DNA extraction Stool Kit® (QIAGEN) according to the mammalian faecal protocol. Briefly, 1 mL of ethanol-fixed faeces was lysed in ASL buffer containing 30 glass beads (4 mm diameter), under permanent stirring conditions. The DNA was thereafter purified using an anion exchange column (QIAGEN) and eluted in 100 *μ*L of distilled water.

For the conventional* G. intestinalis*-PCR, the following specific oligonucleotide sequences were used: the forward oligonucleotide *β*-giardin G7F: 5′-AAGCCCGACGACCTCACCCGCAGTCG-3′ and the reverse oligonucleotide *β*-giardin G759R: 5′-GAGGCCGCCCTGGATCTTCGAGACGAC-3′ [28]. The PCR was performed in a total volume of 25 *μ*L containing 5 *μ*L faecal DNA sample, 5 *μ*L faecal DNA (1 : 100), 1 *μ*L *β*G7F oligonucleotides (10 *μ*M), 1 *μ*L *β*G759R oligonucleotides (10 *μ*M), dNTPs 0.5 *μ*L (10 *μ*M), 0.5 *μ*L Taq-polymerase (1 U/*μ*L; PeqLab), and 14.5 *μ*L H_2_O. The following thermocycle profiles were used: 95°C for 5 min, 35 cycles at 95°C for 30 s, 65°C for 45 s, and 72°C for 1 min and 30 s followed by a final extension step at 72°C for 5 min and a final hold at 20°C. PCR amplificates were visualized in GelRed-stained 2% agarose gels (Biotium Incorporation).

In addition, a* G. intestinalis*-nested PCR was performed. For the nested PCR the following forward oligonucleotide sequences of *β*GiarF were used: 5′-GAACGAGATCGAGGTCCG-3′ and reverse oligonucleotide sequence of *β*GiarR: 5′-CTCGACGAGCTTCGTTGTT-3′ [29]. The following thermocycle profiles for the nested PCR were used: 95°C for 5 min, 35 cycles at 95°C for 30 s, 50°C for 40 s, and 72°C for 1 min and 30 s followed by a final extension step at 72°C for 5 min. PCR amplificates were visualized using GelRed-stained 2% agarose gels as described above (Biotium Incorporation). Further cloning and sequencing were also performed.

## 3. Results

### 3.1. Parasite Infections

**Figure 2:**
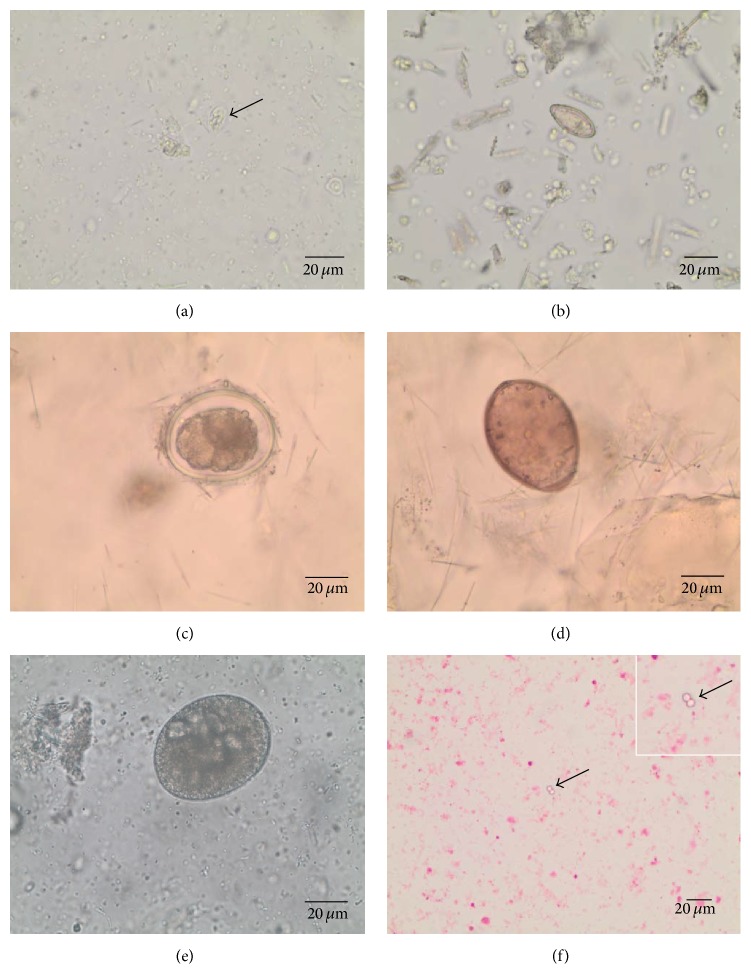
Parasite stages from faecal samples of urban South American sea lions (*Otaria flavescens*): (a)* Giardia* cyst (arrow), (b) Trematoda indet. egg, (c) Anisakidae gen. sp. egg, (d) Diphyllobothriidae gen. sp. egg, (e)* Balantidium *cyst, and (f)* Cryptosporidium* cysts (arrow).

Parasitological analyses of faecal samples of South American sea lions revealed 8 different protozoan (4) and metazoan (4) taxa. The metazoan parasites consisted of trematodes (one species), cestodes (one species), and nematodes (two species). No acanthocephalan parasite eggs were found in the samples. A complete list of the parasite stages and their prevalence is given in Table 1. Illustrations of the parasitic stages are depicted in Figure 2.

The most prevalent metazoan parasites found in this “urban” sea lion colony were Anisakidae gen. sp. (21%) followed by Diphyllobothriidae gen. sp. (13%), Trematoda indet., and* Otostrongylus* sp. larvae (2.5%). The most prevalent protozoan parasites were* Cryptosporidium* (10%),* Giardia*, and* Isospora* showing the same prevalence (5.3%).* Balantidium* infections were detected at lower prevalence (2.5%, Table 1). Within the metazoan endoparasites, the nematodes were the most rich in species (two species) followed by cestodes and trematodes with one species each. Referring to parasite genus level these parasitological findings included four new host records (*Cryptosporidium*,* Giardia*,* Balantidium*, and* Otostrongylus*) for* O. flavescens*. To our best knowledge, the genus* Balantidium* had only been described in fin whales (*Balaenoptera physalus*) from the North Atlantic [27]. All other protozoan parasites have already been reported for other marine mammals [4, 5, 17, 19, 20, 24, 30]. Some of the protozoan (3) and metazoan (2) endoparasite genera detected in sea lions bear an anthropozoonotic potential, such as* Cryptosporidium*,* Giardia*,* Balantidium*, Diphyllobothriidae gen. sp. (*Diphyllobothrium*), and Anisakidae gen. sp. (*Anisakis, Contracaecum, *and* Pseudoterranova*).

### 3.2. Molecular Analyses of Faecal Samples

Although sea lion faecal samples were immediately fixed in 70% ethanol after collection in the field in order to avoid DNA degradation, this goal was not successfully achieved. Thus no adequate* Giardia* DNA was possible to be extracted for further detailed molecular identification.

## 4. Discussion

Common collection methods for analyses of gastrointestinal parasites of wild sea lions generally rely on sections of accidentally stranded animals or on dead animals obtained from marine zoos [5, 14]. Several studies are restricted to the helminth parasite fauna of South American sea lions and include several single species records [4, 5, 15, 16, 18–22, 31]. Thus, protozoan endoparasites have rarely been considered. Conversely, by obtaining fresh faecal samples from resting sea lions, this record reveals unique insights into the actual gastrointestinal parasite fauna of free-ranging sea lions within their natural habitat.

In the present survey, 8 different parasite taxa were detected in individual sea lion faecal samples covering a respectable range of parasitic taxa. The parasitological diagnosis based on morphological criteria revealed as quite a challenge since very little data on parasitic eggs, larvae, cysts and oocysts for sea lions are available in literature. Thus, the photo galleries provided here might supply a supportive tool for future parasitological research activities on sea lions and other marine mammals since many of the parasites described here infect a wide range of marine mammals, such as sea otters, seals, sea elephants, baleen, and toothed cetaceans [30].

The most prevalent parasitic stages found in the current study were eggs of Anisakidae gen. sp. However, owing to undistinguishable egg morphologies, characterization on species level was not possible. Based on parasite frequencies, these eggs most probably belong to the genera* Contracaecum*,* Pseudoterranova*, or* Anisakis* since these parasite species appear to be quite common in South American sea lions [5, 31]. Thus, South American sea lions are known as definitive hosts for the zoonotic nematodes* Anisakis* spp.,* Contracaecum ogmorhini*, and* Pseudoterranova cattani* [5, 31]. Ascarids parasitize either freely in the stomach or firmly attached, often as clusters, to the gastric mucosa [32]. Mucosal penetration via larvae [33] and adults [34] can cause severe ulcers, gastritis, and perforation [32]. Moreover, allergic reactions against epitopes of* Anisakis simplex* major allergen (Ani s1) have been reported to occur in humans after the reexposure to these parasites [35, 36]. The second most prevalent parasite species (13%) was Diphyllobothriidae gen. sp. Consistent to our findings, at least three different diphyllobothriid cestodes have previously been recorded in Chile, two freshwater species (*Diphyllobothrium latum* and* D. dendriticum*) and one marine species (i.e.,* Adenocephalus pacificus* (*Diphyllobothrium pacificum*)) [37, 38] and also in the intestine of stranded sea lions from Patagonia [5], but at a higher prevalence (26.8%). Diphyllobothriasis in sea lions generally is innocuous [32], but debilitation or even death of parasitized hosts might result in cases of high parasitic burdens. In addition, diphyllobothriasis also represents an important fish-borne zoonosis worldwide [39–43].

Overall, low prevalence of trematode and* Otostrongylus* sp. infections (both 2.5%) was diagnosed in the current study. Trematode infections in the pancreas and liver occur in almost all marine mammals by members of the genera* Campula, Zalophotrema, Oschmarinella*, and* Orthosplanchus*. Additionally, also the genera* Apophallus, Ascocotyle, Ogmogaster*, and* Pocitrema* have been reported as intestinal parasites of otariids [5, 44]. These may induce necrosis of the parenchymal tissue, chronic fibrotic hepatitis, enteritis [32], and even meningoencephalitis by aberrant trematode migrations [45]. Furthermore, the trematode genera* Pricetrema* and* Nanophyetes* have been reported to parasitize sea lions in the Northern hemisphere [46]. Whilst* Pricetrema* resides in the liver,* Nanophyetes* infects the small intestine.

Infections with the crenosomatid nematode* Otostrongylus* in pinnipeds are generally associated with respiratory clinical manifestations, primarily in young animals [47]. Adult* Otostrongylus* can obstruct the upper airways causing bronchitis and pneumonitis [47]. Interestingly, some sea lions of the current study showed strong coughing episodes, thereby expelling vast amount of mucus. Based on the current findings this might indicate the presence of clinical otostrongylosis within the Valdivian colony.

The parasitological findings of the study also included four new parasite host reports for* O. flavescens*, namely,* Cryptosporidium, Giardia, Balantidium*, and* Otostrongylus, *thereby providing a broader insight into the spectrum of parasitoses of this marine species. In addition, the protozoan parasites (i.e.,* Cryptosporidium, Giardia, *and* Balantidium*) clearly bear zoonotic potential and are considered as typical water-borne/food-borne diseases. In consequence, the “urban” sea lion colony in Valdivia may function as relevant reservoir for these protozoans since the animals reside at the shore of the river and in close proximity to humans, pet animals, and especially in direct contact to the products of local fish market.

Within the genus* Balantidium, B. coli* is the only species of trichostome ciliates nowadays considered as pathogenic for mammals [48, 49]. Consistent with these findings,* Balatidium* infections in free-ranging fin whales (*Balaenoptera physalus*) from the North Atlantic Ocean were recently diagnosed [30], indicating that this terrestrial disease is circulating in the marine environment. More importantly,* B. coli* infections have been demonstrated in humans and pigs in Chile [50, 51]. As seen for balantidiasis, giardiasis and cryptosporidiosis have an almost cosmopolitan distribution.* Cryptosporidium* and* Giardia* are two common aethiological parasites of infectious enteritis in humans and animals [52, 53]. These enteric protozoans are usually transmitted by the faecal-oral route, following the ingestion of infective stages (oocysts or cysts). Moreover, marine mammals are well known as final hosts of* Giardia* and* Cryptosporidium* [24, 54–58]. Keeping in mind that the urban sea lion colony is close to populated riversides and that some touristic activities, such as “photos with sea lions” or “kayaking with sea lions,” are becoming more popular, sea lions may become infected by human excretions or vice versa. As seen for giardiasis,* Cryptosporidium* infections can cause severe diarrhoea in terrestrial mammals; nonetheless, very little is known on the pathogenesis of cryptosporidiosis within the marine ecosystems [24, 55]. An essential component of the control of these diseases, from a public health perspective, is a better understanding of the sources and routes of transmission in different geographical regions [52].

Unfortunately, the current knowledge and understanding of the ecto- and endoparasite fauna (especially protozoan species) in free-ranging sea lions are still very scarce. Although some parasitoses, such as hookworm (e.g.,* Uncinaria hamiltoni*) or ascarid (*Contracaecum ogmorhini, Pseudoterranova cattani*, and* Anisakis* spp.) [5], infections are discussed as pathogenic species for sea lions [32], the total parasite fauna of marine vertebrates has unfortunately not obtained sufficient attention, so far [24, 59].

In conclusion, this study adds some new anthropozoonotic parasite records to free-ranging sea lions and calls for more integrated research to avoid the exposure of humans and domestic animals with these parasites. In particular, regular monitoring programs should be established by local authorities for public health issues and for sea lion populations living in close proximity to human beings.

## Figures and Tables

**Table 1:** Prevalence (in percentage) of parasitic infections in South American sea lions (*Otaria flavescens*), technique, and sample origin.

	Parasites	(%)	Technique	Material
Metazoan parasites	Anisakidae gen. sp.	21	SAF	Faeces
	Diphyllobothriidae gen. sp.	13	SAF	Faeces
	Trematoda indet.	2.5	SAF	Faeces
	*Otostrongylus *sp.	2.5	SAF	Faeces

Protozoan parasites	*Cryptosporidium*	10	CFS/coproELISA	Faeces
	*Giardia*	5.3	SAF/coproELISA	Faeces
	*Isospora*	5.3	SAF	Faeces
	*Balantidium *	2.5	SAF	Faeces
